# Diabetes Mellitus among Adult Tuberculosis Patients Attending Tuberculosis Clinics in Eastern Ethiopia

**DOI:** 10.1155/2019/7640836

**Published:** 2019-11-06

**Authors:** Lucy Tenaye, Bizatu Mengiste, Negga Baraki, Ermiyas Mulu

**Affiliations:** ^1^College of Health Sciences and Medicine, Haramaya University, Harar, Ethiopia; ^2^College of Medicine and Health Sciences, Ambo University, Ambo, Ethiopia

## Abstract

**Background:**

Developing countries are suffering from the previously existing infectious diseases and alarmingly growing burden of noncommunicable diseases like diabetes mellitus. There is increased speculation that diabetes mellitus might attribute to high infectious diseases burden, such as tuberculosis. The global importance of diabetes mellitus as a tuberculosis-risk factor is still not a well-established fact. Thus, we conducted this study to determine the prevalence of diabetes mellitus and its associated factors among adult tuberculosis patients attending tuberculosis clinics.

**Methodology:**

We conducted a cross-sectional survey, from March 10 to April 15, 2017, among 421 tuberculosis patients receiving tuberculosis treatment in health facilities of Dire Dawa City Administration Council, Eastern Ethiopia. Study participants were selected using systematic random technique, and data were collected using a structured questionnaire. Fasting blood sugar and anthropometric measurements were carried out for all participants. A logistic regression analysis was performed to identify factors associated with diabetes mellitus.

**Result:**

The prevalence of diabetes mellitus in this study was 13.5%. Age 26–40 (AOR = 6, 95% CI: (1.28, 27.5)), age ≥41(AOR = 9, 95% CI: (1.9, 44.4)), and family history of diabetes (AOR = 3.14, 95% CI: (1.23, 8.02)) were found to have a significant association with diabetes mellitus.

**Conclusion:**

This study found that the magnitude of diabetes mellitus among tuberculosis patients was higher than the national estimated prevalence of diabetes mellitus in Ethiopia. This study suggests the need for screening each tuberculosis patient for diabetes.

## 1. Background

DM (diabetes mellitus) is defined as a cluster of metabolic disorders, characterized by hyperglycemia high enough to significantly increase the incidence of a specific and unique type of microangiopathy (retinopathy, nephropathy, and neuropathy) [1]. Tuberculosis (TB) is an infectious disease caused by various strains of mycobacterium, especially *Mycobacterium tuberculosis* and usually attacks the lung [2].

Diabetic patients have the evidence of impaired cell-mediated immunity, micronutrient deficiency, pulmonary microangiopathy, and renal insufficiency, all of which predispose to pulmonary tuberculosis (PTB) [3, 4]. The stress of a severe chronic infection may enhance existing insulin resistance and unmask an underlying *β*-cell deficiency leading to hyperglycemia; therefore it is possible that the risk of DM is increased among people with TB, especially in the presence of other predisposing factors [5].

Increases in the burden of noncommunicable diseases and aging populations are changing the importance of different risk factors for tuberculosis and the profile of comorbidities and clinical challenges for people with tuberculosis [6]. Early detection and prompt controlling are important for diabetes mellitus and tuberculosis. Nevertheless, the early diagnosis of diseases is less common in developing countries. The originally existing disease masks the symptoms of the complicating disease. The prognosis and clinical course of each of the two conditions adversely affect the other [7].

The relationship and the merging epidemics of tuberculosis (TB) and diabetes mellitus (DM) in populations with low socioeconomic status have raised concerns among many experts [8]. Low-income countries, such as in Ethiopia, are facing double burden of alarming rise in DM prevalence and the highest burden of TB in the world. The possible link between the two diseases will further complicate the problem and seek special concern [9, 10].

Several studies have shown that DM increases the risk of TB and that patients with TB have higher rates of DM [11–13]. Diabetes triples the risk of tuberculosis and is also a risk factor for adverse tuberculosis treatment outcomes, including death [14]. These risks are known to become worse in people living with DM, especially if their blood glucose levels are high [15, 16].

Given the high prevalence of both DM and TB in Ethiopia, it is likely that many patients have comorbidity [17, 18]. However, the problem of diabetes in TB patients of Ethiopia is not well documented; although there are pocket studies conducted in a localized area, these pocket studies indicated that problem of diabetes among TB patients is increasing. Therefore, this study aims to assess the diabetes mellitus comorbidity among patients on tuberculosis treatment.

## 2. Methods

### 2.1. Study Setting

We conducted a cross-sectional study from March 10 to April 15, 2017, in all TB clinics of Dire Dawa city Administrative Council, Eastern Ethiopia. A total of 463 TB patients were selected, using systematic random sampling technique, from 25 public and private health facilities that were providing TB treatment service in the city.

### 2.2. Data Collection Procedure

The study used both primary (structured questionnaire, anthropometric measurement, and fasting blood glucose test) and secondary (TB patient smear status, TB treatment category, and type of TB) data collection techniques. Data on sociodemographic and clinical characteristics were collected by interviewing the patients using a structured and pretested questionnaire. The questionnaire was prepared in English and translated into Amharic questions, Afan Oromo, and Somali language (languages spoken by study participants). To check the consistency and clarity of the items in the tool, all versions (Amharic, Afan Oromo, and Somali language) of the questionnaire were pretested on 23 TB patients attending the TB clinic at Hiwotfana Hospital.

All patients diagnosed as having active TB were screened for DM through their history, previous medical records, and measurement of fasting blood glucose (FBG) concentrations. The glucometer (Prodigy auto code) device was used for the determination of fasting plasma glucose level. Blood sugar status was classified based on the American Diabetes Association cutoff points. Accordingly, patients were classified into three groups: normal (70–99 m/dL), prediabetes (100–125 mg/dL), and diabetes (≥126 mg/dL) [19]. Height and weight of the patients were measured using a digital weight scale and stadiometer. BMI was calculated, and weight status was classified using the WHO cutoff points.

Data collectors and supervisors were trained on the objectives and methodology of the research, data collection and interviewing approach, anthropometric measurement, glucose measurement, and data recording. At least one blood glucose measurement (FPG) was carried out for each patient. In the case of (FPG) ≥ 126 mg/dL, a second determination was performed one week later. Two height and weight measurements were taken from each participant, and procedures were repeated when two measurements did not yield the required precision.

### 2.3. Data Processing and Analysis

We checked the completeness and consistency of each questionnaire. Then, data were sorted, coded, and entered into a computer using EpiData. After being cleaned by checking for error, impossible or implausible values, and inconsistencies, the data were transferred and analyzed using SPSS version 20 software. The results of the descriptive analysis were summarized using cross tabulation and descriptive statistics. The correlates of DM were assessed using binary logistic regression analysis. Odds ratio with 95% confidence interval was calculated to measure the strength of association between dependent and independent variables. To control possible confounders, assess factors significantly associated with diabetes and variables associated with *p* < 0.3 in the univariable analysis were entered into the multivariable logistic analysis model. Statistical associations were asserted based on 95% CI and two-sided 5% level of significance (*α* < 0.05).

## 3. Results

### 3.1. Diabetes Mellitus

Of the total 463 intended, 430 (92.87%) patients consented and underwent screening. Thirty-three (7.8%) patients refused to participate in the screening. Nine (1.9%) patients were excluded from the analysis, as they did not have a confirmatory result for DM. As a result, 421 (90.92%) participants were included in the final analysis.

**Figure 1:**
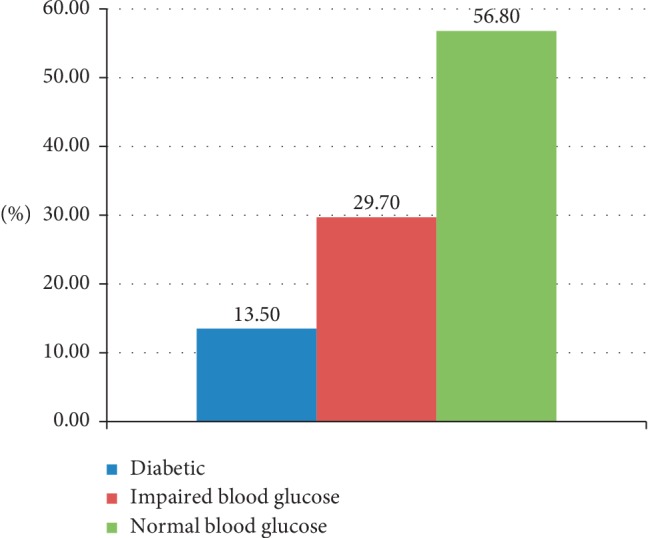
Fasting plasma glucose level of TB patients.

Our FPG assessment showed that about 13.5% of TB patients had DM comorbidity (FPG ≥ 126 mg/dL) and about one-third (29.7%) of TB patients had impaired fasting plasma glucose value (FPG 100–125 mg/dL) (Figure 1). About half of diabetic comorbid (52.7%) was known patients while the remaining were newly diagnosed diabetic patients. Nearly one in twelve diabetic individuals reported that they had a family history of diabetes mellitus.

### 3.2. Sociodemographic and Clinical Characteristics

The mean age of the participants was 34.08 (±15.126) years, and males were the majority (55.8%). Most of the study participants (90.9%) were urban dwellers. About three-fourths of the patients had attended at least primary education (Table 1).

Three-hundred thirty-eight (80.3%) of the study participants were PTB patients, and of these, one-hundred eighty-eight (44.6%) were smear-positive. Majority of (14.2%) of TBDM patients were PTB Patients. The overall prevalence of chronic energy deficiency and overweight of TB patients was 40.8 and 6.4%, respectively. It seems that the risk of diabetes comorbidity increases by twofold for normal weight and fourfold for overweight TB patients compared with their underweight counterparts (Table 2).

### 3.3. Factors Associated with Diabetes Mellitus among TB Patients

We conducted a binary logistic regression analysis to identify the factors correlated with DM among patients on TB treatment. In the univariable analysis; age, education, occupation, and family history of diabetes had a significant statistical association (*p* < 0.05) with DM. A multivariable direct logistic regression analysis was performed to detect factors significantly associated with DM. Independent variables that showed significant association at *p* < 0.3 were entered into the final model (multivariable logistic analysis). The model contained nine independent variables (sex, age, marital status, occupation, education, family history, BMI, TB treatment category, and type of TB).

As indicated in Table 3, only age and family history of diabetes made a unique statistically significant contribution to the model. The age of the patients was a strong predictor of diabetes mellitus, recording odds ratio of 9 (≥41 years) and 6 (26–40 years old). These indicate that TB patients older than 41 years were 9 times (AOR = 9.74, 95% CI: (2.69, 20.6)) and between 26 and 40 years were 6 times (AOR = 6, 95% CI: 1.28, 27.5) more likely to be diabetic compared to younger age (between 18 and 25 years) TB patients. TB patients who have a family history of diabetes is 3 times (AOR = 3.14, 95% CI: (1.23, 8.02)) more diabetic compared with those who had no family history of diabetes.

## 4. Discussion

The prevalence of DM among patients with TB in Eastern Ethiopia was higher than the national estimated prevalence of DM [17, 20]. Similar studies conducted in India and Mexico reported a higher prevalence of diabetes mellitus comorbidity among TB than our finding [11, 21]. On the other hand, studies carried out in Nigeria (1.9%), China (3.2%), Ethiopia (8.5%), Tanzania (7.81%), and Uganda (8.5%) revealed a lower prevalence of DM among TB patients than our finding [5, 22–25]. The wide range of prevalence of DM in different studies might be due to the difference in sociodemographic characteristics of source populations in the localities studied and screening methods used in DM diagnosis.

Type II diabetes mellitus and other lifestyle-related chronic diseases are more prevalent in old age. In this study, most of the DM-positive individuals were greater than or equal to 41 years of age. When age is increased, the prevalence of DM also increases, which was consistent with the studies done in Ethiopia and Kerala, India [11, 13, 23, 26, 27]. Increasing age is linked to immune suppression and is one of the risk factors for both TB and DM [28].

Diabetes is a disease which has strong clustering in families, and family history of DM is a risk factor for DM [29–31]. The result of our study demonstrated this fact; the prevalence of DM was higher among patients who have a family history of DM. This finding is supported by the shreds of evidence from other studies conducted in Ethiopia and other developing countries [13, 25, 32]. This fact might suggest the gap and necessity of revising the existing screen and management approach, individual, of diabetes mellitus.

The proportion of new DM patients 27(6.4%) identified in our study was higher than the figures documented by similar studies from China (3%) and Ethiopia [22, 33]. A study from India revealed a higher proportion of new DM (9.3%) among TB patients [13]. Development of Type II DM is a gradual process. Symptomatic type II DM is a result of unnoticeable body metabolism alterations in years. Prediabetes and diabetes screening is important to reduce the risk of developing diabetes and manage the disease in early stages. The high proportion of new DM patients among our study participants indicates low diabetes screening coverage in TB clinics [34]. Therefore, the finding of this research calls for the implementation of active case finding of DM in patients diagnosed for TB and also for the integrating of TB and DM care programs.

In this study, the overall proportion of prediabetes among TB patients was 29.7%, which is consistent with a similar study performed in Gonder, Ethiopia [35]. Prediabetes is a condition characterized by abnormally high blood glucose but lower than the diabetic range. Prediabetes individuals have a higher risk of developing DM; each year about 5% to 10% of prediabetes individuals develop DM [36]. Provided low diabetes screening coverage and health promotion activity in Ethiopia, the current number of DM patients may surge significantly in the near future [35]. The observed DM and prevalence of prediabetes in the studied group warrants integrated health services approach to address the burden of the two diseases.

The authors acknowledge that exclusion of uncertain first blood glucose results will be a possible limitation of this study. A significant number of TB patients, whose first DM test result was not conclusive to determine the DM status, were excluded from the analysis. As a result, we may have underestimated the prevalence of DM among TB patients.

## 5. Conclusion

The magnitude of DM among TB patients in the study settings, Dire Dawa, was higher than the national estimated prevalence of DM in Ethiopia. Age and family history of diabetes were found to have significant association with diabetes mellitus. About half of the DM patients in this study were diagnosed for the first time during this study. Therefore, the concerned body should give attention to implementing diabetes screening and comprehensive chronic care at TB clinics as it might have a beneficial impact on TB control and management of diabetes mellitus. Advance studies are needed for further understanding of the relationships and management approaches of TB-DM comorbidity.

## Figures and Tables

**Table 1:** Sociodemographic characteristics of TB patients.

Variables	Nondiabetic	Diabetic	Total
*Sex*			
Male	207 (88.1%)	28 (12%)	235 (55.8%)
Female	157 (84.4%)	29 (15.6%)	186 (44.2%)

*Marital status*			
Single	171 (95%)	9 (5%)	180 (42.7%)
Married	175 (80.6%)	42 (19.35%)	217 (51.5%)
Divorced	9 (69.2%)	4 (30.78%)	13 (3.08%)
Widowed	9 (81.8%)	2 (18.2%)	11 (2.6%)

*Age in year*			
18–25	137 (96.5%)	5 (3.5%)	142 (33.7%)
26–40	134 (85.4%)	23 (14.6%)	157 (37.3%)
≥41	93 (76.2%)	29 (23.8%)	122 (29%)

*Residence*			
Urban	331 (86.4%)	52 (13.6%)	383 (90.9%)
Rural	33 (86.8%)	5 (13.2)	38 (9.02%)

*Educational status*			
No formal education	88 (78.6%)	24 (21.4%)	112 (26.6%)
Elementary school	102 (89.5%)	12 (10.5%)	114 (27.1%)
Secondary school	139 (89.7%)	16 (10.3%)	155 (36.8%)
Higher education	35 (87.5%)	5 (12.5%)	40 (9.5%)

*Occupational status*			
House wife	49 (80.3%)	12 (19.7%)	61 (14.5%)
Unemployed	56 (82.3%)	12 (17.6%)	68 (16.1%)
Daily labour	47 (79.6%)	12 (20.3%)	59 (14%)
Merchant	87 (91.6%)	8 (8.4%)	95 (22.6%)
Government employee	64 (86.5%)	10 (13.5%)	74 (17.6%)
Others	61 (95.3%)	3 (4.7%)	64 (15.2%)

*Income*			
<1000	83 (85.6%)	14 (14.4%)	97 (23%)
1000–2000	150 (85.2%)	26 (14.7%)	176 (41.8%)
2001–3000	66 (85.7%)	11 (14.3%)	77 (18.3%)
>3000	65 (91.5%)	6 (8.45%)	71 (16.8%)

**Table 2:** Clinical characteristics and lifestyle of TB patients by diabetes mellitus.

Variables	TB patients	TB patients with DM	Total
*BMI*			
<18.5	157 (91.3%)	15 (8.7%)	172 (40.8%)
18.5–24.9	188 (85.1%)	33 (15%)	221 (52.5%)
25–29.9	16 (76.2%)	5 (23.8%)	21 (5%)
≥30	2 (33.3%)	4 (66.7%)	6 (1.4%)

*Family history of DM*			
Yes	23 (69.7%)	10 (30.3%)	33 (7.84%)
No	256 (88%)	35 (12%)	291 (69.1%)
Not known	85 (87.6%)	12 (12.4%)	97 (23%)

*Chat chewing*			
Yes	145 (87.4)	21 (12.6%)	166 (39.4%)
No	219 (85.8)	36 (14.1%)	255 (60.6%)

*Cigarette smoking*			
Never smoke	293 (87.2%)	43 (12.8%)	336 (79.8%)
Primary cigars smoke	26 (83.8%)	5 (16.1%)	31 (7.4%)
Ex cigars smoker	40 (83.3%)	8 (16.7%)	48 (11.4%)
Secondary cigar smoker	5 (83.3%)	1 (16.7%)	6 (1.4%)

*TB treatment category*			
Category 1	308 (87.7%)	43 (12.25%)	351 (83.4%)
Category 2	56 (80%)	14 (20%)	70 (16.6%)

*Type of TB*			
Smear +ve PTB	157 (91.3%)	31 (16.5%)	188 (44.6%)
Smear –ve PTB	133 (88.7%)	17 (11.3%)	150 (35.6%)
EPTB	74 (89.2%)	9 (10.8%)	83 (19.7%)

DM = diabetes mellitus; EPTB = extrapulmonary tuberculosis; PTB = pulmonary tuberculosis; TB = tuberculosis.

**Table 3:** Factors associated with diabetes mellitus among patients tuberculosis on treatment, Eastern Ethiopia.

Variable	Category	TB comorbid with DM		COR(95% CI)	AOR(95% CI)
		Yes	No		
Sex	Male	28 (12%)	207 (88.1%)	0.73 (0.42, 1.3)	0.515 (0.25–1.16)
	Female	29 (15.6)	157 (84.4%)		

Age	18–25	5 (3.5%)	137 (96.5%)		
	26–40	23 (14.6%)	134 (85.4%)	4.703 (1.74, 12.7)^*∗*^	6 (1.282–27.5)^*∗*^
	≥41	29 (23.8%)	93 (76.2%)	8.544 (3.2, 22.8)^*∗*^	9 (1.9–44.4)^*∗*^

Marital status	Single	15 (7.4%)	189 (92.6%)		
	Married	42 (19.4%)	175 (80.6%)	0.331 (0.18, 0.617)	1.5 (0.692–3.41)

Occupational status	House wife	12 (19.7%)	49 (80.3%)	1.57 (0.63, 3.925)	0.7 (0.184–2.74)
	Unemployed	12 (17.6%)	56 (82.3%)	1.4 (0.551, 3.42)	1.3 (0.380–4.4)
	Daily labour	12 (20.3%)	47 (79.6%)	1.63 (0.651, 4.1)	1.62 (0.5–5.31)
	Merchant	8 (8.4%)	87 (91.6%)	0.59 (0.22–1.575)	0.52 (0.152–1.75)
	Government	10 (13.5%)	64 (86.5%)		
	Others	3 (4.7%)	61 (95%)	0.315 (0.08–1.2)	2.3 (0.32–17.4)

Educational status	Have no formal education	24 (21.4%)	88 (78.6%)	1.91 (0.675, 5.402)	1.7 (0.366–7.450)
	Primary/secondary	12 (10.5%)	102 (89.5%)	0.8 (0.29–2.245)	1.03 (0.28–3.8)
	Higher education	16 (10.3%)	139 (89.7%)		

Family history of diabetes	Yes	10 (30.3%)	23 (69.7%)	3.154 (1.4, 7.04)^*∗*^	3.14 (1.232–8.02)^*∗*^
	No	47 (12%)	341 (88%)		

BMI	<18.5	15 (8.7%)	157 (91.3%)		
	18.5–24.9	33 (15%)	188 (85.1%)	1.87 (0.98, 3.57)	1.95 (0.97–3.9)
	≥25	9 (33.3%)	18 (66.7%)	4.74 (1.836, 12.24)^*∗*^	2.72 (0.911–8.105)

TB treatment category	Category 1	43 (12.25%)	308 (87.7%)		
	Category 2	14 (20%)	56 (80%)	1.791 (0.92, 3.5)	0.56 (0.261–1.2)

Type of TB	Smear +ve PTB	31 (16.5%)	157 (91.3%)	1.6 (0.735, 3.6)	2.31 (0.87–6.1)
	Smear –ve PTB	17 (11.3%)	133 (88.7%)	1.05 (0.45, 2.5)	1.6 (0.575–4.4)
	EPTB	9 (10.8%)	74 (89.2%)		

^*∗*^Significant at*p* < 0.05; DM = diabetes mellitus; EPTB = extrapulmonary tuberculosis; PTB = pulmonary tuberculosis; TB = tuberculosis.

## Data Availability

The full data used to support the findings of this study are available at the hands of the first author upon request.

## Abbreviations

ADA: American Diabetic Association
BMI: Body mass index
CI: Confidence interval
DM: Diabetes mellitus
EPTB: Extrapulmonary tuberculosis
FBG: Fasting blood glucose
FPG: Fasting plasma glucose
mg/dL: milligram/deciliter
HIV: Human immunodeficiency virus
IDF: International Diabetes Federation
IFG: Impaired fasting glucose
IGT: Impaired glucose tolerance
MTB: *Mycobacterium tuberculosis*
NCDs: Noncommunicable diseases
OGTT: Oral glucose tolerance test
PTB: Pulmonary tuberculosis
RBS: Random blood sugar
TB: Tuberculosis
WHO: World Health Organization.
